# Crystal structure of 1-(4-meth­oxy­phen­yl)-4-(4-nitro­phen­yl)-3-phen­oxy­azetidin-2-one

**DOI:** 10.1107/S2056989014025833

**Published:** 2015-01-01

**Authors:** Sevim Türktekin Çelikesir, Mehmet Akkurt, Aliasghar Jarrahpour, Habib Allah Shafie, Ömer Çelik

**Affiliations:** a Department of Physics, Faculty of Sciences, Erciyes University, 38039 Kayseri, Turkey; b Department of Chemistry, College of Sciences, Shiraz University, 71454 Shiraz, Iran; c Department of Physics, Faculty of Education, Dicle University, 21280, Diyarbakir, Turkey; d Science and Technology Application and Research Center, Dicle University, 21280, Diyarbakir, Turkey

**Keywords:** crystal structure, phen­oxy­azetidin-2-one, β-lactam ring, four-membered monocyclic aza-heterocycles, anti­biotics, C—H⋯O hydrogen bonds, C—H⋯π inter­actions

## Abstract

In the title compound, C_22_H_18_N_2_O_5_, the central β-lactam ring (r.m.s. deviation = 0.002 Å) makes dihedral angles of 64.21 (14), 82.35 (12) and 20.66 (13)° with the phenyl ring and the nitro- and meth­oxy­benzene rings, respectively. The mol­ecular structure is stabilized by an intra­molecular C—H⋯O hydrogen bond. In the crystal, mol­ecules are linked *via* C—H⋯O hydrogen bonds, forming slabs lying parallel to (111). The slabs are linked *via* C—H⋯π inter­actions, forming a three-dimensional network.

## Related literature   

For general properties and applications in medicinal chemistry of four-membered monocyclic aza-heterocycles, see: Bode *et al.* (1989 ▸); Gerona-Navarro *et al.* (2004 ▸); Grafe (1992 ▸); Gérard *et al.* (2004 ▸); Mehta *et al.* (2010 ▸); Setti *et al.* (2005 ▸); Singh *et al.* (2008 ▸); Southgate (1994 ▸); Sutton *et al.* (2004 ▸); Sperka *et al.* (2005 ▸). For related structures, see: Akkurt *et al.* (2011 ▸); Butcher *et al.* (2011 ▸).
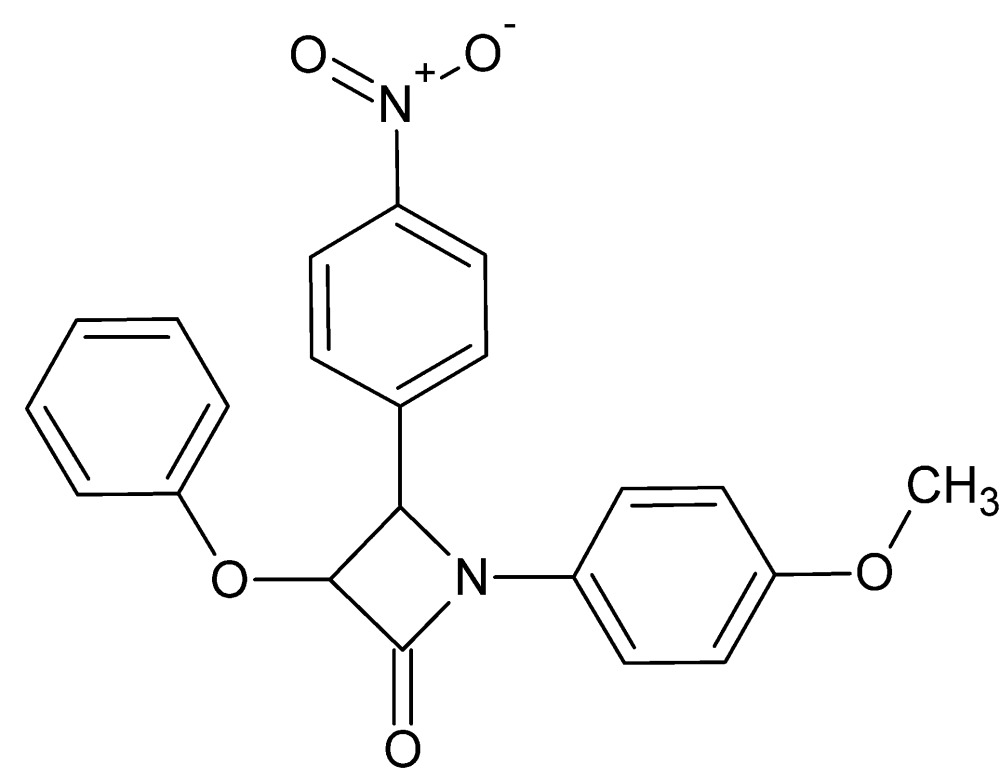


## Experimental   

### Crystal data  

C_22_H_18_N_2_O_5_
*M* *_r_* = 390.38
Triclinic, 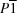

*a* = 9.8044 (3) Å
*b* = 10.6483 (3) Å
*c* = 11.1573 (3) Å
α = 66.957 (1)°
β = 70.105 (1)°
γ = 65.973 (1)°
*V* = 956.06 (5) Å^3^
*Z* = 2
Mo *K*α radiation
μ = 0.10 mm^−1^
*T* = 296 K
0.30 × 0.20 × 0.15 mm

### Data collection  

Bruker APEXII CCD diffractometer
16870 measured reflections
3586 independent reflections
2860 reflections with *I* > 2σ(*I*)
*R*_int_ = 0.027

### Refinement  

*R*[*F* ^2^ > 2σ(*F* ^2^)] = 0.046
*wR*(*F* ^2^) = 0.125
*S* = 1.09
3586 reflections
257 parameters
H-atom parameters constrained
Δρ_max_ = 0.22 e Å^−3^
Δρ_min_ = −0.17 e Å^−3^

### 

Data collection: *APEX2* (Bruker, 2007 ▸); cell refinement: *SAINT* (Bruker, 2007 ▸); data reduction: *SAINT*; program(s) used to solve structure: *SHELXS2014* (Sheldrick, 2008 ▸); program(s) used to refine structure: *SHELXL2014* (Sheldrick, 2008 ▸); molecular graphics: *ORTEP-3 for Windows* (Farrugia, 2012 ▸); software used to prepare material for publication: *PLATON* (Spek, 2009 ▸).

## Supplementary Material

Crystal structure: contains datablock(s) global, I. DOI: 10.1107/S2056989014025833/su5026sup1.cif

Structure factors: contains datablock(s) I. DOI: 10.1107/S2056989014025833/su5026Isup2.hkl

Supporting information file. DOI: 10.1107/S2056989014025833/su5026Isup3.cml

**Fig. 1.**
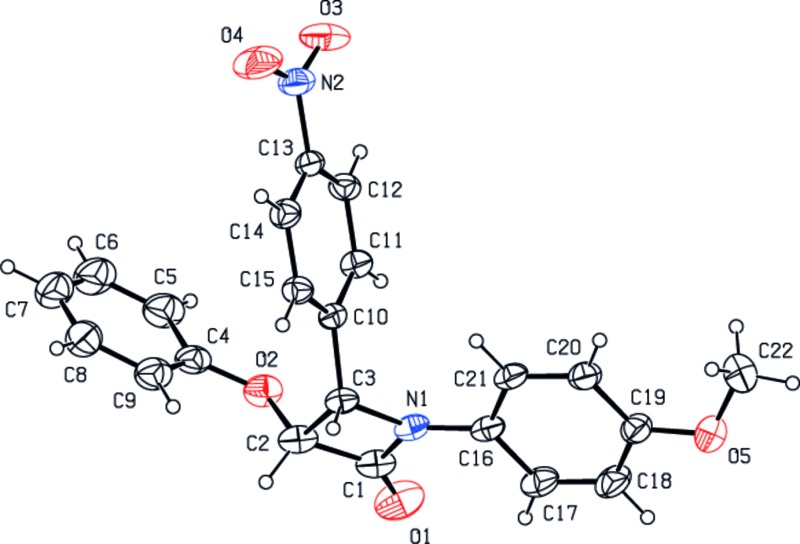
Perspective view of the molecular structure of the title compound, with atom labelling. Displacement ellipsoids are drawn at the 30% probability level.

**Fig. 2.**
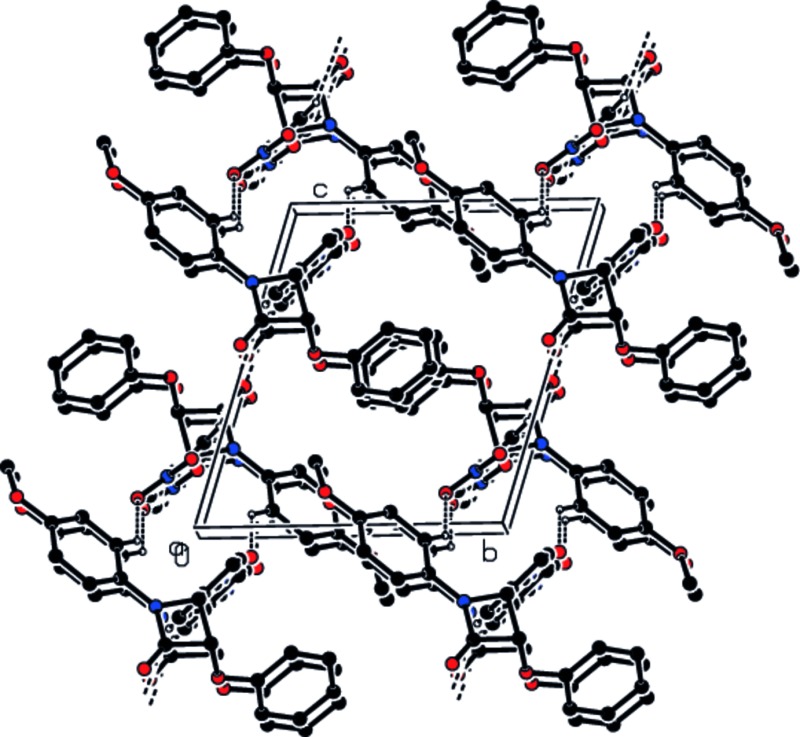
View of the hydrogen bonding and molecular packing of the title compound along a axis (only H atoms involved in hydrogen bonding are shown; see Table 1 for details).

. DOI: 10.1107/S2056989014025833/su5026fig1.tif
Perspective view of the mol­ecular structure of the title compound, with atom labelling. Displacement ellipsoids are drawn at the 30% probability level.

a . DOI: 10.1107/S2056989014025833/su5026fig2.tif
View of the hydrogen bonding and mol­ecular packing of the title compound along *a* axis (only H atoms involved in hydrogen bonding are shown; see Table 1 for details).

CCDC reference: 1036033

Additional supporting information:  crystallographic information; 3D view; checkCIF report

## Figures and Tables

**Table 1:** Hydrogen-bond geometry (, ) *Cg*4 is the centroid of the methoxyphenyl ring (C16C21).

*D*H*A*	*D*H	H*A*	*D* *A*	*D*H*A*
C17H17O1	0.93	2.60	3.172(3)	120
C12H12O1^i^	0.93	2.37	3.103(2)	135
C21H21O4^ii^	0.93	2.43	3.127(3)	132
C15H15*Cg*4^iii^	0.93	2.75	3.674(2)	173

Symmetry codes: (i) 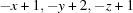; (ii) 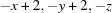; (iii) 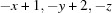.

## supplementary crystallographic information

### S1. Comment

Four-membered monocyclic aza-heterocycles (Singh, *et al.*, 2008), even more than 70 years after the discovery of penicillin, β-lactam antibiotics remain as one of the most important contributions of science to humanity (Southgate, 1994). β-Lactam antibiotics have been successfully used in the treatment of infectious diseases for many years (Grafe, 1992). Literature survey reveals that 2-azetidinones show to possess other relevant biological activities (Gerona-Navarro *et al.*, 2004). which include human cytomegalovirus (HCMV) inhibitor, (Mehta *et al.*, 2010), human leukocyte elastase (HLE) inhibitor, (Gérard *et al.*, 2004), thrombin inhibitor, (Sutton *et al.*, 2004), porcine pancreatic elastase (PPE) inhibitor, (Bode *et al.*, 1989), HIV-1 protease inhibitor (Sperka *et al.*, 2005), cysteine protease inhibitor (Setti *et al.*, 2005).

In the title compound (Fig. 1), the β-lactam ring (N1/C1–C3) is nearly planar [r.m.s. deviation = 0.002 Å]. It makes dihedral angles of 64.21 (14), 82.35 (12) and 20.66 (13)° with the phenyl ring (C4–C9) and the nitro- and methoxybenzene rings (C10–C15 and C16–C21), respectively.

The bond lengths and bond angles are normal and are similar to the corresponding bond distances and angles reported for similar compound, *viz*. 1-(4-methoxyphenyl)-4-(4-methylphenyl)-3-phenoxyazetidin-2-one (Akkurt *et al.*, 2011) and 3-(4-chlorophenoxy)-1-(4-methoxyphenyl)-4-(4-nitrophenyl)azetidin-2-one (Butcher *et al.*, 2011).

A weak intramolecular C—H···O hydrogen bond stabilizes the molecular conformation (Table 1).

In the crystal, molecules are linked by C—H···O hydrogen bonds forming slabs lying parallel to (111). The slabs are linked via C—H···π interactions forming a three dimensional network (Table 1 and Fig. 2).

### S2. Experimental

A solution of (*E*)-4-methoxy-*N*-(4-nitrobenzylidene)aniline (1.00 mmol) was stirred with the phenoxy acetic acid (1.50 mmol), *p*-toluenesulfonyl chloride (1.50 mmol) and triethylamine (5.0 mmol) in dry CH_2_Cl_2_ at room temperature overnight. Then it was washed with HCl 1 N (20 ml), saturated NaHCO_3_ (20 ml), brine (20 ml), dried over Na_2_SO_4_ and the solvent was evaporated under reduced pressure to give the crude product. It was then recrystallized from hexan/EtOAc (2:6) to give colourless prisms (yield 75%; m.p: 413–415 K). IR (KBr, cm^-1^): 1744 (CO, β-lactam). ^1^H-NMR (250 MHz CDCl_3_), δ (p.p.m.): 3.69 (OMe, s, 3H), 5.01 (H-4, d, 1H, J = 4.7 HZ), 5.40 (H-3, d, 1H, J = 4.7 HZ), 6.69–8.10 (ArH, m, 13H). ^13^C-NMR (62.9 MHz, CDCl_3_), δ (p.p.m.): 54.3 (OMe), 63.7 (C-4), 83.1 (C-3), 114.5–157.2 (aromatic carbons), 162.8 (CO, β-lactam). GC—MS m/*z* = 391 [*M*+]. Analysis calculated for C_22_H_18_N_2_O_5_: C, 67.69; H, 4.65; N, 7.18%. Found: C, 67.65; H, 4.70; N, 7.20%.

### S3. Refinement

All H atoms were placed in calculated positions, with C—H = 0.93 - 0.98 Å, and refined using a riding model with *U*_iso_(H) = 1.5*U*_eq_(C) for methyl H atoms and = 1.2U_eq_(C) for other H atoms. The crystal was of very low quality and in the final cycles of refinement 45 reflections were omitted owing to very bad agreement [reflections (-6 7 5), (-6 7 3), (-5 8 5), (-6 7 2), (-5 8 4), (-5 8 2), (-5 8 3), (-4 9 3), (-4 8 3), (-5 7 1), (-5 7 2), (-6 6 2), (-4 8 5), (-5 8 1), (-4 8 2), (-5 7 4), (-4 9 4), (-5 7 0), (-6 7 4), (-5 7 3), (-4 9 2), (-6 5 1), (-6 6 3), (-4 8 1), (-5 6 3), (-5 6 2), (-4 8 4), (0 10 2), (-5 6 0), (-6 6 1), (-4 7 4), (-6 6 0), (-4 7 0), (-4 7 3), (-4 7 1), (-3 8 4), (-3 8 3), (-4 7 2), (6 - 5 1), (-5 6 1), (-6 4 0), (6 - 6 1), (0 - 4 2), (5 - 6 1), (1 1 1)].

### Crystal data

**Table:**

C_22_H_18_N_2_O_5_	*Z* = 2
*M**_r_* = 390.38	*F*(000) = 408
Triclinic, *P*1	*D*_x_ = 1.356 Mg m^−^^3^
Hall symbol: -P 1	Mo *K*α radiation, λ = 0.71073 Å
*a* = 9.8044 (3) Å	Cell parameters from 8412 reflections
*b* = 10.6483 (3) Å	θ = 2.2–31.2°
*c* = 11.1573 (3) Å	µ = 0.10 mm^−^^1^
α = 66.957 (1)°	*T* = 296 K
β = 70.105 (1)°	Prism, colourless
γ = 65.973 (1)°	0.30 × 0.20 × 0.15 mm
*V* = 956.06 (5) Å^3^	

### Data collection

**Table:**

Bruker APEXII CCD diffractometer	2860 reflections with *I* > 2σ(*I*)
Radiation source: sealed tube	*R*_int_ = 0.027
Graphite monochromator	θ_max_ = 26.4°, θ_min_ = 2.0°
φ and ω scans	*h* = −12→12
16870 measured reflections	*k* = −13→13
3586 independent reflections	*l* = −13→13

### Refinement

**Table:**

Refinement on *F*^2^	0 restraints
Least-squares matrix: full	Hydrogen site location: inferred from neighbouring sites
*R*[*F*^2^ > 2σ(*F*^2^)] = 0.046	H-atom parameters constrained
*wR*(*F*^2^) = 0.125	*w* = 1/[σ^2^(*F*_o_^2^) + (0.054*P*)^2^ + 0.2069*P*] where *P* = (*F*_o_^2^ + 2*F*_c_^2^)/3
*S* = 1.09	(Δ/σ)_max_ < 0.001
3586 reflections	Δρ_max_ = 0.22 e Å^−^^3^
257 parameters	Δρ_min_ = −0.17 e Å^−^^3^

### Special details

**Table:**

Geometry. Bond distances, angles *etc*. have been calculated using the rounded fractional coordinates. All su's are estimated from the variances of the (full) variance-covariance matrix. The cell e.s.d.'s are taken into account in the estimation of distances, angles and torsion angles
Refinement. Refinement on *F*^2^ for ALL reflections except those flagged by the user for potential systematic errors. Weighted *R*-factors *wR* and all goodnesses of fit *S* are based on *F*^2^, conventional *R*-factors *R* are based on *F*, with *F* set to zero for negative *F*^2^. The observed criterion of *F*^2^ > σ(*F*^2^) is used only for calculating -*R*-factor-obs *etc*. and is not relevant to the choice of reflections for refinement. *R*-factors based on *F*^2^ are statistically about twice as large as those based on *F*, and *R*-factors based on ALL data will be even larger.

### Fractional atomic coordinates and isotropic or equivalent isotropic displacement parameters (Å^2^)

**Table:**

	*x*	*y*	*z*	*U*_iso_*/*U*_eq_	
O1	0.19496 (17)	1.0034 (2)	0.46300 (14)	0.0891 (6)	
O2	0.50869 (15)	0.76274 (16)	0.48302 (11)	0.0619 (5)	
O3	1.15569 (17)	0.9287 (2)	0.20735 (17)	0.0938 (7)	
O4	1.22134 (17)	0.7927 (2)	0.08655 (19)	0.1013 (8)	
O5	0.20258 (17)	1.58682 (16)	−0.07766 (16)	0.0783 (6)	
N1	0.38058 (14)	1.03204 (17)	0.26380 (13)	0.0488 (5)	
N2	1.12538 (18)	0.86578 (19)	0.15529 (16)	0.0629 (6)	
C1	0.3090 (2)	0.9617 (2)	0.38519 (16)	0.0569 (5)	
C2	0.42887 (19)	0.8176 (2)	0.37976 (16)	0.0569 (5)	
C3	0.50356 (17)	0.90236 (19)	0.23890 (14)	0.0450 (5)	
C4	0.6280 (2)	0.6349 (2)	0.48961 (17)	0.0575 (7)	
C5	0.7040 (3)	0.5907 (3)	0.5903 (2)	0.0768 (9)	
C6	0.8249 (3)	0.4657 (3)	0.6043 (3)	0.0928 (10)	
C7	0.8714 (3)	0.3834 (3)	0.5201 (3)	0.0896 (10)	
C8	0.7957 (3)	0.4277 (3)	0.4207 (2)	0.0775 (8)	
C9	0.6737 (2)	0.5537 (2)	0.40422 (19)	0.0663 (7)	
C10	0.66584 (17)	0.89768 (17)	0.21679 (14)	0.0394 (5)	
C11	0.70598 (18)	0.95456 (19)	0.28763 (15)	0.0459 (5)	
C12	0.85691 (19)	0.94417 (19)	0.26790 (15)	0.0477 (5)	
C13	0.96466 (17)	0.87828 (18)	0.17624 (15)	0.0448 (5)	
C14	0.92900 (19)	0.82065 (19)	0.10398 (16)	0.0491 (5)	
C15	0.77834 (18)	0.83228 (18)	0.12446 (15)	0.0452 (5)	
C16	0.33669 (17)	1.1729 (2)	0.17642 (16)	0.0473 (6)	
C17	0.1869 (2)	1.2640 (2)	0.1967 (2)	0.0628 (7)	
C18	0.1477 (2)	1.4003 (2)	0.1106 (2)	0.0687 (8)	
C19	0.2549 (2)	1.4496 (2)	0.0030 (2)	0.0569 (7)	
C20	0.4030 (2)	1.3594 (2)	−0.01778 (18)	0.0540 (6)	
C21	0.44279 (18)	1.2218 (2)	0.06850 (17)	0.0505 (6)	
C22	0.3131 (3)	1.6499 (3)	−0.1745 (3)	0.0853 (10)	
H2	0.38870	0.74890	0.37620	0.0680*	
H3	0.49120	0.87820	0.16780	0.0540*	
H5	0.67340	0.64540	0.64820	0.0920*	
H6	0.87620	0.43620	0.67200	0.1120*	
H7	0.95330	0.29860	0.53050	0.1070*	
H8	0.82660	0.37250	0.36340	0.0930*	
H9	0.62310	0.58320	0.33610	0.0800*	
H11	0.63100	0.99990	0.34870	0.0550*	
H12	0.88460	0.98110	0.31580	0.0570*	
H14	1.00460	0.77530	0.04320	0.0590*	
H15	0.75150	0.79570	0.07560	0.0540*	
H17	0.11350	1.23260	0.26840	0.0750*	
H18	0.04730	1.46060	0.12470	0.0820*	
H20	0.47610	1.39090	−0.08980	0.0650*	
H21	0.54290	1.16120	0.05350	0.0610*	
H22A	0.38120	1.65160	−0.13090	0.1280*	
H22B	0.26230	1.74640	−0.22200	0.1280*	
H22C	0.37030	1.59430	−0.23600	0.1280*	

### Atomic displacement parameters (Å^2^)

**Table:**

	*U*^11^	*U*^22^	*U*^33^	*U*^12^	*U*^13^	*U*^23^
O1	0.0574 (9)	0.1347 (15)	0.0630 (8)	−0.0226 (9)	0.0108 (7)	−0.0452 (9)
O2	0.0622 (8)	0.0815 (10)	0.0467 (6)	−0.0293 (8)	−0.0155 (6)	−0.0135 (6)
O3	0.0683 (10)	0.1387 (16)	0.1022 (11)	−0.0573 (11)	−0.0252 (8)	−0.0319 (11)
O4	0.0394 (8)	0.1325 (16)	0.1246 (14)	−0.0241 (10)	0.0052 (9)	−0.0532 (13)
O5	0.0615 (9)	0.0556 (10)	0.1099 (11)	−0.0048 (8)	−0.0253 (8)	−0.0250 (8)
N1	0.0310 (7)	0.0673 (11)	0.0485 (7)	−0.0124 (7)	−0.0042 (5)	−0.0244 (7)
N2	0.0428 (9)	0.0751 (12)	0.0627 (9)	−0.0265 (9)	−0.0142 (7)	−0.0017 (8)
C1	0.0444 (7)	0.0846 (10)	0.0485 (6)	−0.0267 (6)	−0.0063 (5)	−0.0231 (7)
C2	0.0444 (7)	0.0846 (10)	0.0485 (6)	−0.0267 (6)	−0.0063 (5)	−0.0231 (7)
C3	0.0373 (8)	0.0606 (12)	0.0440 (8)	−0.0166 (8)	−0.0094 (6)	−0.0207 (7)
C4	0.0511 (11)	0.0701 (14)	0.0490 (9)	−0.0322 (11)	−0.0124 (8)	−0.0003 (9)
C5	0.0813 (15)	0.0930 (18)	0.0630 (11)	−0.0416 (15)	−0.0289 (11)	−0.0038 (11)
C6	0.0886 (18)	0.101 (2)	0.0842 (16)	−0.0368 (17)	−0.0482 (14)	0.0097 (15)
C7	0.0692 (15)	0.0826 (18)	0.0955 (17)	−0.0272 (14)	−0.0310 (13)	0.0099 (14)
C8	0.0671 (14)	0.0733 (16)	0.0806 (14)	−0.0245 (13)	−0.0168 (11)	−0.0079 (11)
C9	0.0632 (12)	0.0766 (15)	0.0600 (11)	−0.0293 (12)	−0.0187 (9)	−0.0090 (10)
C10	0.0349 (8)	0.0431 (10)	0.0399 (7)	−0.0114 (7)	−0.0105 (6)	−0.0109 (6)
C11	0.0400 (9)	0.0550 (11)	0.0461 (8)	−0.0132 (8)	−0.0083 (7)	−0.0211 (7)
C12	0.0481 (9)	0.0544 (11)	0.0488 (8)	−0.0215 (9)	−0.0169 (7)	−0.0123 (7)
C13	0.0349 (8)	0.0463 (10)	0.0471 (8)	−0.0154 (8)	−0.0127 (6)	−0.0017 (7)
C14	0.0397 (9)	0.0520 (11)	0.0502 (8)	−0.0119 (8)	−0.0041 (7)	−0.0170 (8)
C15	0.0420 (9)	0.0516 (11)	0.0470 (8)	−0.0158 (8)	−0.0082 (7)	−0.0197 (7)
C16	0.0325 (8)	0.0626 (12)	0.0554 (9)	−0.0097 (8)	−0.0105 (7)	−0.0308 (8)
C17	0.0358 (9)	0.0778 (15)	0.0706 (11)	−0.0106 (10)	−0.0028 (8)	−0.0327 (11)
C18	0.0370 (10)	0.0707 (15)	0.0918 (14)	0.0017 (10)	−0.0103 (9)	−0.0401 (12)
C19	0.0453 (10)	0.0519 (13)	0.0806 (12)	−0.0058 (9)	−0.0210 (9)	−0.0303 (10)
C20	0.0401 (9)	0.0593 (13)	0.0666 (10)	−0.0137 (9)	−0.0116 (8)	−0.0247 (9)
C21	0.0304 (8)	0.0616 (13)	0.0609 (9)	−0.0083 (8)	−0.0098 (7)	−0.0261 (9)
C22	0.0902 (17)	0.0591 (15)	0.1103 (18)	−0.0270 (13)	−0.0321 (14)	−0.0162 (13)

### Geometric parameters (Å, º)

**Table:**

O1—C1	1.199 (3)	C14—C15	1.376 (3)
O2—C2	1.413 (2)	C16—C17	1.391 (3)
O2—C4	1.382 (3)	C16—C21	1.379 (3)
O3—N2	1.208 (3)	C17—C18	1.372 (3)
O4—N2	1.208 (3)	C18—C19	1.385 (3)
O5—C19	1.372 (3)	C19—C20	1.376 (3)
O5—C22	1.421 (4)	C20—C21	1.383 (3)
N1—C1	1.369 (2)	C2—H2	0.9800
N1—C3	1.468 (3)	C3—H3	0.9800
N1—C16	1.414 (2)	C5—H5	0.9300
N2—C13	1.469 (3)	C6—H6	0.9300
C1—C2	1.515 (3)	C7—H7	0.9300
C2—C3	1.570 (2)	C8—H8	0.9300
C3—C10	1.508 (3)	C9—H9	0.9300
C4—C5	1.382 (3)	C11—H11	0.9300
C4—C9	1.377 (3)	C12—H12	0.9300
C5—C6	1.371 (4)	C14—H14	0.9300
C6—C7	1.373 (4)	C15—H15	0.9300
C7—C8	1.367 (4)	C17—H17	0.9300
C8—C9	1.384 (4)	C18—H18	0.9300
C10—C11	1.388 (3)	C20—H20	0.9300
C10—C15	1.388 (2)	C21—H21	0.9300
C11—C12	1.384 (3)	C22—H22A	0.9600
C12—C13	1.370 (2)	C22—H22B	0.9600
C13—C14	1.379 (3)	C22—H22C	0.9600
			
C2—O2—C4	118.48 (15)	C18—C19—C20	119.10 (19)
C19—O5—C22	117.6 (2)	C19—C20—C21	119.87 (19)
C1—N1—C3	95.42 (14)	C16—C21—C20	121.16 (19)
C1—N1—C16	133.12 (17)	O2—C2—H2	113.00
C3—N1—C16	130.07 (13)	C1—C2—H2	113.00
O3—N2—O4	123.0 (2)	C3—C2—H2	113.00
O3—N2—C13	118.75 (18)	N1—C3—H3	111.00
O4—N2—C13	118.26 (19)	C2—C3—H3	111.00
O1—C1—N1	132.1 (2)	C10—C3—H3	111.00
O1—C1—C2	135.63 (18)	C4—C5—H5	120.00
N1—C1—C2	92.29 (15)	C6—C5—H5	120.00
O2—C2—C1	110.35 (15)	C5—C6—H6	119.00
O2—C2—C3	117.28 (17)	C7—C6—H6	120.00
C1—C2—C3	85.76 (13)	C6—C7—H7	120.00
N1—C3—C2	86.47 (13)	C8—C7—H7	120.00
N1—C3—C10	117.56 (16)	C7—C8—H8	120.00
C2—C3—C10	117.93 (14)	C9—C8—H8	120.00
O2—C4—C5	115.15 (19)	C4—C9—H9	120.00
O2—C4—C9	124.79 (18)	C8—C9—H9	120.00
C5—C4—C9	120.1 (2)	C10—C11—H11	120.00
C4—C5—C6	119.5 (2)	C12—C11—H11	120.00
C5—C6—C7	121.1 (3)	C11—C12—H12	121.00
C6—C7—C8	119.2 (3)	C13—C12—H12	121.00
C7—C8—C9	120.9 (2)	C13—C14—H14	121.00
C4—C9—C8	119.3 (2)	C15—C14—H14	121.00
C3—C10—C11	121.92 (15)	C10—C15—H15	119.00
C3—C10—C15	118.78 (16)	C14—C15—H15	119.00
C11—C10—C15	119.29 (18)	C16—C17—H17	120.00
C10—C11—C12	120.36 (16)	C18—C17—H17	120.00
C11—C12—C13	118.56 (17)	C17—C18—H18	119.00
N2—C13—C12	118.97 (17)	C19—C18—H18	119.00
N2—C13—C14	118.34 (16)	C19—C20—H20	120.00
C12—C13—C14	122.69 (18)	C21—C20—H20	120.00
C13—C14—C15	118.03 (17)	C16—C21—H21	119.00
C10—C15—C14	121.06 (17)	C20—C21—H21	119.00
N1—C16—C17	121.10 (16)	O5—C22—H22A	109.00
N1—C16—C21	120.06 (18)	O5—C22—H22B	109.00
C17—C16—C21	118.83 (18)	O5—C22—H22C	109.00
C16—C17—C18	119.84 (19)	H22A—C22—H22B	109.00
C17—C18—C19	121.2 (2)	H22A—C22—H22C	110.00
O5—C19—C18	116.24 (19)	H22B—C22—H22C	109.00
O5—C19—C20	124.65 (19)		
			
C2—O2—C4—C5	177.80 (19)	C2—C3—C10—C15	115.35 (17)
C4—O2—C2—C1	−176.84 (16)	N1—C3—C10—C11	37.9 (2)
C4—O2—C2—C3	−81.0 (2)	C2—C3—C10—C11	−63.5 (2)
C2—O2—C4—C9	−1.7 (3)	O2—C4—C5—C6	−179.5 (2)
C22—O5—C19—C18	169.3 (2)	O2—C4—C9—C8	179.7 (2)
C22—O5—C19—C20	−12.1 (3)	C9—C4—C5—C6	0.0 (4)
C16—N1—C1—C2	169.0 (2)	C5—C4—C9—C8	0.2 (3)
C3—N1—C1—O1	−178.4 (2)	C4—C5—C6—C7	−0.2 (5)
C16—N1—C1—O1	−11.3 (4)	C5—C6—C7—C8	0.2 (5)
C1—N1—C16—C21	170.0 (2)	C6—C7—C8—C9	0.0 (5)
C16—N1—C3—C10	70.7 (2)	C7—C8—C9—C4	−0.2 (4)
C3—N1—C16—C21	−26.9 (3)	C3—C10—C11—C12	177.87 (15)
C16—N1—C3—C2	−169.53 (19)	C11—C10—C15—C14	1.2 (2)
C3—N1—C1—C2	1.90 (16)	C15—C10—C11—C12	−1.0 (2)
C1—N1—C3—C2	−1.84 (15)	C3—C10—C15—C14	−177.68 (15)
C3—N1—C16—C17	152.68 (19)	C10—C11—C12—C13	0.7 (3)
C1—N1—C16—C17	−10.4 (3)	C11—C12—C13—C14	−0.7 (3)
C1—N1—C3—C10	−121.65 (15)	C11—C12—C13—N2	−179.56 (16)
O4—N2—C13—C14	−7.7 (3)	N2—C13—C14—C15	179.77 (16)
O3—N2—C13—C14	172.35 (18)	C12—C13—C14—C15	0.9 (3)
O4—N2—C13—C12	171.23 (18)	C13—C14—C15—C10	−1.2 (3)
O3—N2—C13—C12	−8.8 (3)	N1—C16—C21—C20	−179.51 (18)
N1—C1—C2—O2	115.78 (16)	C17—C16—C21—C20	0.9 (3)
O1—C1—C2—C3	178.6 (3)	N1—C16—C17—C18	179.76 (18)
N1—C1—C2—C3	−1.78 (15)	C21—C16—C17—C18	−0.6 (3)
O1—C1—C2—O2	−63.9 (3)	C16—C17—C18—C19	0.0 (3)
O2—C2—C3—C10	10.4 (2)	C17—C18—C19—C20	0.4 (3)
O2—C2—C3—N1	−109.09 (17)	C17—C18—C19—O5	179.1 (2)
C1—C2—C3—C10	121.13 (17)	O5—C19—C20—C21	−178.7 (2)
C1—C2—C3—N1	1.66 (14)	C18—C19—C20—C21	−0.1 (3)
N1—C3—C10—C15	−143.22 (15)	C19—C20—C21—C16	−0.5 (3)

### Hydrogen-bond geometry (Å, º)

Cg4 is the centroid of the methoxyphenyl ring (C16–C21).

**Table:**

*D*—H···*A*	*D*—H	H···*A*	*D*···*A*	*D*—H···*A*
C17—H17···O1	0.93	2.60	3.172 (3)	120
C12—H12···O1^i^	0.93	2.37	3.103 (2)	135
C21—H21···O4^ii^	0.93	2.43	3.127 (3)	132
C15—H15···*Cg*4^iii^	0.93	2.75	3.674 (2)	173

Symmetry codes: (i) −*x*+1, −*y*+2, −*z*+1; (ii) −*x*+2, −*y*+2, −*z*; (iii) −*x*+1, −*y*+2, −*z*.
