# Supplementary figures and images for: Crystal structure of 1-(4-meth­oxy­phen­yl)-4-(4-nitro­phen­yl)-3-phen­oxy­azetidin-2-one
Source: Acta Crystallogr E Crystallogr Commun. 2015 Jan 1;71(Pt 1):o12–3. doi: 10.1107/S2056989014025833 (PMC4331848; doi:10.1107/S2056989014025833)

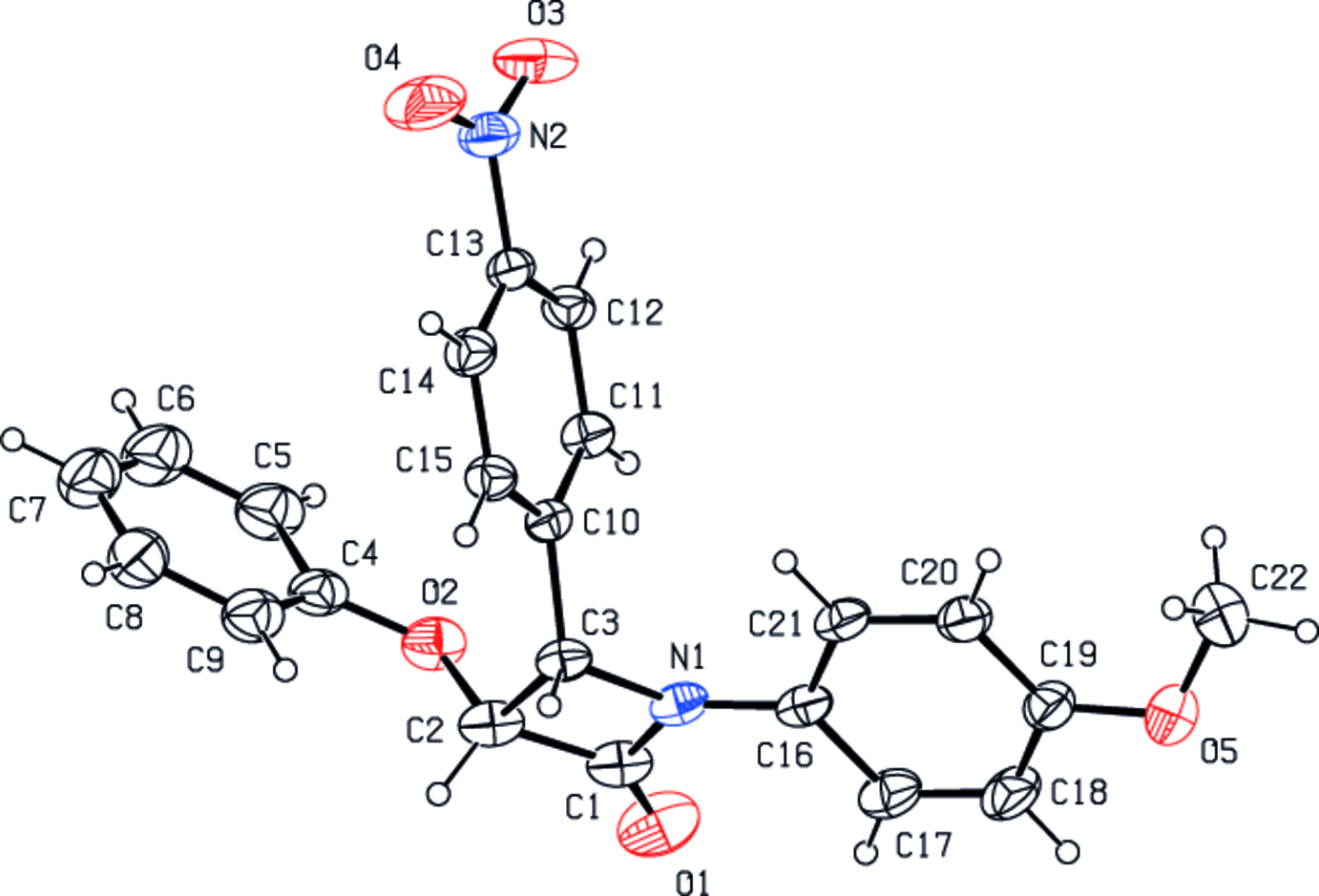

Supplement: Supplementary file 4 [file e-71-00o12-fig1.tif]

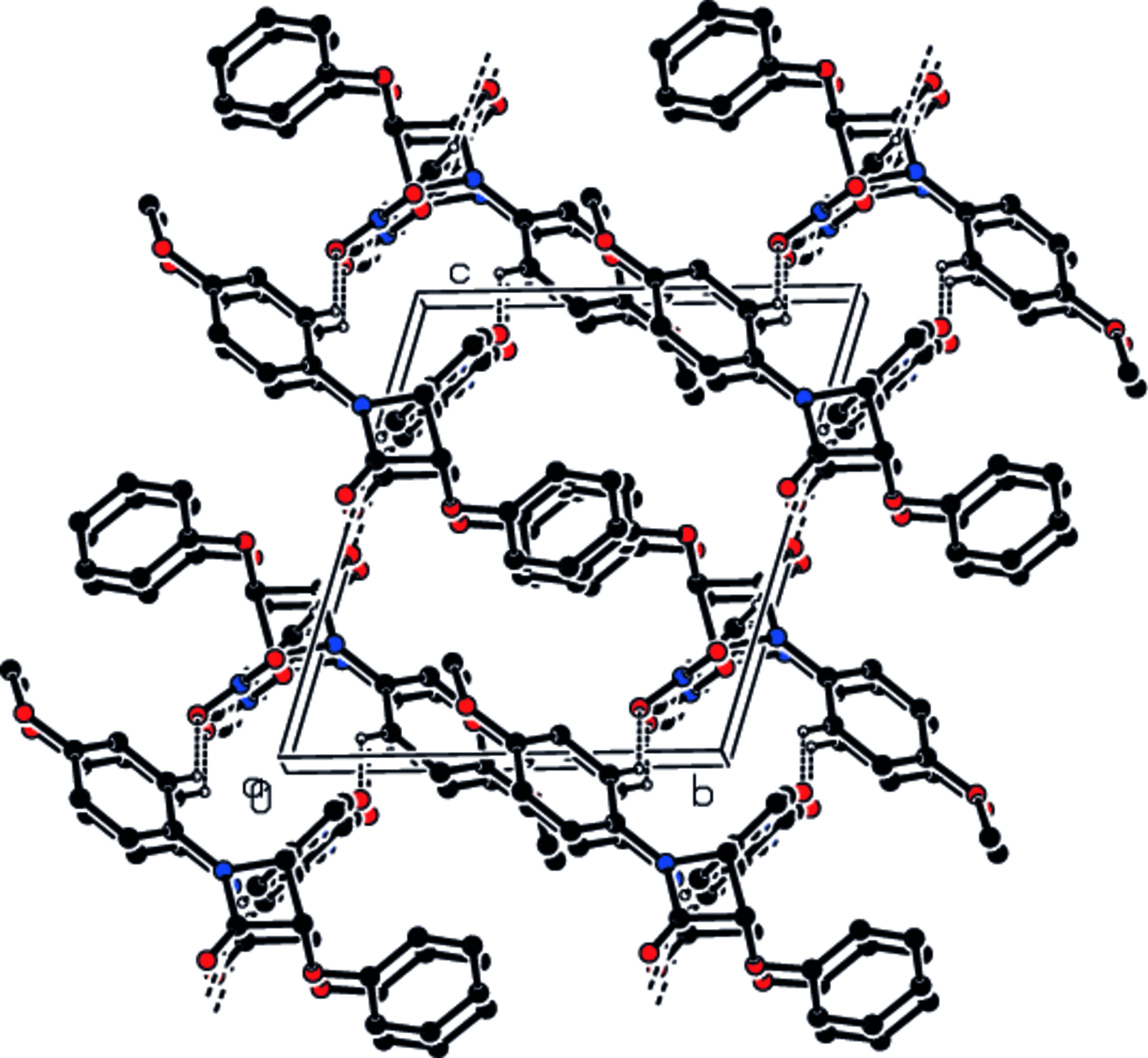

Supplement: Supplementary file 5 [file e-71-00o12-fig2.tif]
